# How Food Geometry and Toughness Influence Ingestive Patterns in Lemurs

**DOI:** 10.1002/ajp.70188

**Published:** 2026-07-12

**Authors:** Mariana Dutra Fogaça, Nina Flowers, Haja Fabrice Razafindrabe Maminiaina, Jean Claude Razafimampiandra, Nayuta Yamashita

**Affiliations:** ^1^ Institute of Population Genetics University of Veterinary Medicine Vienna Austria; ^2^ Neotropical Primates Research Group ‐ NeoPReGo São Paulo Brazil; ^3^ KWR Water Research Institute Nieuwegein PE The Netherlands; ^4^ Department of Zoology and Animal Biodiversity University of Antananarivo Antananarivo Madagascar; ^5^ Austrian Academy of Sciences Vienna Austria

**Keywords:** bite location, food mechanical properties, ingestion, *Lemur catta*, *Propithecus verreauxi*, localização da mordida, propriedades mecânicas dos alimentos, ingestão

## Abstract

We investigate the extent to which food toughness, geometry, and/or lemur species determine biting locations during feeding. We quantified biting locations in two sympatric species, *Lemur catta* and *Propithecus verreauxi*, in Beza Mahafaly Special Reserve, Madagascar. Using video of seven lemur groups, we assigned biting locations to anterior, intermediate, or posterior positions. Food toughness (average and maximum) was measured using a portable field tester. Foods were categorized into four shape (flat, not‐flat) and size (small, large) categories. Multinomial logistic regression models were used to test these predictors with food placement as the response. Toughness and food geometry significantly influenced biting positions, though not necessarily as predicted. *P. verreauxi* favored anterior placements for small, non‐flat foods, whereas *L. catta* favored intermediate, and both used posterior with increasing food toughness. *P. verreauxi* has a robust masticatory system and can process “moderate” tough food anteriorly (initially defaulting to this position). However, they process food posteriorly as the food increases in toughness. *L. catta* more evenly distributes bites among the ingestion locations. These two lemur species differ in initial feeding behaviors that are likely related to underlying differences in masticatory morphology.

## Graphical abstract

Initial bite placement is influenced by food toughness and geometry. Species‐specific feeding strategies reflect differences in masticatory morphology and functional flexibility. *Lemur catta* is more likely to use the postcanine tooth row; *Propithecus verreauxi* prefers anterior biting.

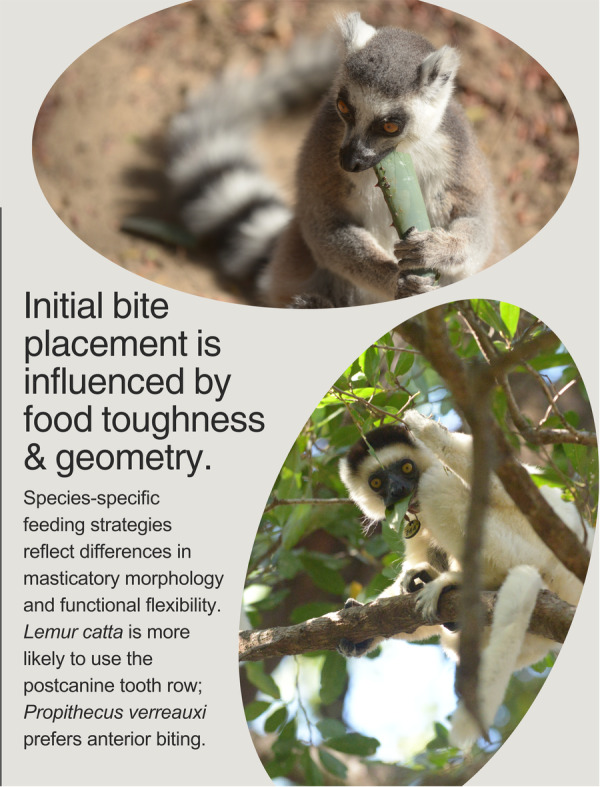

## Resumo

Investigamos em que medida a geometria, a resistência à fratura do alimento, e as variações interespecíficas dos lêmures influenciam o local inicial de mordida. Quantificamos o posicionamento inicial do alimento para duas espécies simpátricas de lêmures (*Lemur catta* e *Propithecus verreauxi*) na Reserva Especial Beza Mahafaly, Madagascar. Categorizamos o posicionamento inicial dos alimentos: anterior (ANT), intermediária (INT) ou posterior (POST). A resistência à fratura dos alimentos (média e máxima) foi medida no campo, usando um equipamento portátil. Os alimentos foram categorizados em quatro categorias de forma (planos e não planos), em tamanho (pequeno e grande). Testamos a probabilidade de posicionamento inicial do alimento com os preditores acima, utilizando modelos de regressão logística multinomial. A resistência à fratura máxima e a geometria do alimento influenciam significativamente o lugar da mordida, embora não necessariamente como previsto. *P. verreauxi* favoreceu a colocação de alimentos anteriormente para alimentos pequenos e não planos, enquanto *L. catta* preferiu colocação em posição intermediária e ambos usaram mais os posteriores para alimentos com maior resistencia a fratura. O *P. verreauxi*, mais robusto, processa alimentos moderadamente resistentes anteriormente, no entanto, à medida que a resistencia a fratura aumenta, recorrem à região posterior. *L. catta* distribui as mordidas de maneira mais uniforme entre os locais de ingestão. Essas duas espécies de lêmures diferem nos comportamentos alimentares, o que provavelmente está relacionado a diferenças subjacentes na morfologia mastigatória.

## 1 Introduction

Feeding behavior is known to play a role in the evolution of the masticatory apparatus in extant primates (e.g., Daegling 1992; Fabre et al. 2018; López‐Aguirre et al. 2022; Ross and Iriarte‐Diaz 2014), and fossil hominids (e.g., Strait et al. 2009); however, the influence of specific behaviors, such as biting, on morphological variation (e.g., jaw joint configuration, architecture and attachment of masticatory muscles, tooth form and cusp structure) remains poorly understood.

In vivo studies have demonstrated that patterns of stress, strain, and deformation in the craniofacial skeleton are fundamental to interpreting primate craniofacial anatomy (Ross et al. 2016). Such laboratory studies have shown that biting produces variable loads and strains, for example, linking feeding behavior to craniodental morphology in the platyrrhine, *Sapajus*, in which ingestive behaviors (biting and oral food preparation) result in higher mandibular strains than mastication (Ross et al. 2016). Likewise, finite element analyses have demonstrated that strain varies along the toothrow, providing insights into how biting at different locations influences stress in the facial skeleton (Cox et al. 2015; Dumont et al. 2005).

These studies underscore the need to investigate food handling and oral processing behaviors in wild populations (e.g., Coiner‐Collie et al. 2016; McGraw and Daegling 2019; Ross et al. 2012, 2016). Ingestive behavior can enable primates to process mechanically challenging foods beyond their nominal bite‐force capacity; for instance, the cercopithecine, *Cercocebus atys*, is able to open the seed casings of *Sacoglottis* sp. despite the tough shell requiring forces beyond the species’ bite force capability (Daegling et al. 2011). They achieve this through ingestive behavioral strategies (isometric biting) that enable them to process these seeds without relying on excessively high bite forces (Daegling et al. 2011).

While the cases above illustrate that behavior can compensate for mechanical constraints, they also raise important questions about the extent to which masticatory traits themselves constrain or enable behavioral flexibility in feeding. Among extant primates, for example, while the cercopithecines, *Cercopithecus* and *Lophocebus*, have similar mandibular robusticity, *Cercopithecus* consumes harder seeds using the postcanines, and *Lophocebus* relies on the anterior dentition (Daegling and McGraw 2007; McGraw et al. 2011). Similarly, although the eight species of *Sapajus* have similar craniodental features, the mechanical properties of their diets can vary significantly (Chalk et al. 2016; Wright 2005). Different species and individual sex/age categories (e.g., female, male, juvenile) handle mechanically challenging foods in different ways (e.g., Chalk et al. 2016; Rose 1994; Sabbatini et al. 2008; Williamson et al. 2021), indicating that feeding behavior can diverge even within similar morphological frameworks. These studies illustrate the complexity of the interactions between masticatory morphology and ingestion, especially with respect to how primates manage mechanically demanding foods. This topic requires further exploration.

Understanding feeding behavior requires consideration of both the location of biting along the toothrow and the temporal patterns of these behaviors, as they influence how force is applied to food. The location of biting along the toothrow and consideration of the frequency and duration of these behaviors are critical for efficiently processing food and managing stress during feeding. Studies in humans and other animals have revealed variations in force produced at different points along the toothrow (e.g., in lizards, Anderson et al. 2008; hyenas, Binder and Van Valkenburgh 2000; bats, Dumont and Herrel 2003; humans, Edmonds and Glowacka 2020; monkeys, Laird et al. 2023; opossums, Thompson et al. 2003). Among primates, *Chiropotes*, known for having a mechanically challenging diet, uses the anterior teeth to breach the seed coat (e.g., *Licania majuscula*) and masticates the relatively soft kernel on the postcanines (Martin et al. 2003; Kinzey and Norconk 1990, 1993). Similarly, *Sapajus libidinosus* processes stiffer and tougher foods anteriorly rather than on the postcanines (Laird et al. 2020), whereas *S. apella* uses the first and second molars for breaking open seed casings (Terborgh 1983; Wright 2005), even though the anterior dentition of the species may be better able to resist high stresses on the mandible (Daegling 1992). Collectively, this implies that choosing the ideal position for exerting force on food may be a crucial decision that facilitates the effectiveness of feeding.

Food size is another important factor that influences bite location. If an individual consumer does not break food into pieces before ingesting it, the larger the food, the wider the gape (the extent to which the mouth is opened during ingestion). However, bite force and gape are inversely related (Herring and Herring 1974); the wider the gape, the weaker the force produced. Maximum gape size significantly influences feeding behaviors in strepsirrhines, with frugivores (and their larger gapes) consistently selecting prepared food blocks of larger size than folivores (Perry and Hartstone‐Rose 2010). Folivores also produce greater bite force at smaller gapes than frugivores due to their shorter jaws, shorter adductor muscle fibers, and larger adductor muscle physiological cross‐sectional areas (Perry et al. 2011b). This sets up a potential conflict between processing foods that require a large gape versus foods that require greater bite force, as well as foods that require both. Certain traits, such as increased muscle excursion found in *Macaca fascicularis* and *Callithrix jacchus*, however, can enable wide jaw opening without compromising force generation or performance (Eng et al. 2009; Terhune et al. 2011).

Furthermore, food mechanical properties (FMPs; e.g., elastic modulus, toughness) and structural geometry (food shape and size) also influence the distribution of stresses and strains in feeding systems, thereby affecting both immediate mechanical performance and long‐term structural changes (e.g., Ravosa et al. 2015; Taylor et al. 2008). These food properties can also guide where food is initially positioned on the jaw, reflecting behavioral adjustments that occur prior to jaw loading (Yamashita 2003; Yamashita et al. 2009). However, quantification of these properties is not straightforward. The complex external and internal morphology of plants (e.g., irregular cross‐sections, nodes, and branches) and their anisotropic, non‐linear, and time‐dependent mechanical responses (Zhang et al. 2024) make obtaining standardized measures of mechanical properties challenging (Shah et al. 2017). The development of the FLS‐I, a portable mechanical tester, has enabled field testing of dietary mechanical properties and has been widely adopted in field studies (e.g., Coiner‐Collier et al. 2016; Chalk et al. 2016; Flowers et al. 2023; Lucas et al. 2001; Lucas 2004; Yamashita 2003; Yamashita et al. 2009, 2024).

Two sympatric primate species in southwestern Madagascar further illustrate how species can possess different morphologies and behaviors within a shared environment. Comparative analyses of *Lemur catta* and *Propithecus verreauxi* reveal distinct cranio‐dental and masticatory morphologies. *L. catta* exhibits a more gracile skull with a lower zygomatic arch (Kay et al. 1978) and an unfused mandibular symphysis (linked to low bite force production; Perry et al. 2011a). In contrast, *P. verreauxi* has a robust skull with a high zygomatic arch, high‐cusped molars with distinctive crests (e.g., Cuozzo and Yamashita 2006), and a deep mandibular corpus and ramus with partial symphyseal fusion, features consistent with generating a high bite force (Groves and Helgen 2007; Hylander et al. 2011; Knigge et al. 2020; Ravosa and Vinyard 2020). In addition, *L. catta* possesses a lower mandibular condyle and a longer jaw (Perry et al. 2011a) and relatively longer fascicles of the jaw muscles than *Propithecus* (Dickinson et al. 2024), traits that are associated with wider gapes in gouging primates (Vinyard et al. 2003) and used as markers of gape capacity in living and fossil taxa (Perry et al. 2015; Seiffert et al. 2009). These features suggest *L. catta* likely achieves a wider gape but produces lower bite forces than *P. verreauxi*. The evolution of the masticatory apparatus reflects the effects of maximum food toughness and the cumulative strain from daily biting and chewing in *L. catta* and *P. verreauxi* (Flowers et al. 2023; Yamashita et al. 2024). This suggests that the shape of the masticatory apparatus in these species is more closely related to biting and oral preparation than previously expected, and that the lemurs’ use of the toothrow should be further investigated. For example, in Yamashita (2003), the placement of foods on the toothrow was determined by food geometry as much as by food toughness.

The current study, conducted at Beza Mahafaly Special Reserve (BMSR) in Madagascar, examines the initial placement of foods on the toothrow during oral processing with respect to food toughness and external properties. The two lemur study species are the ring‐tailed lemur and Verreaux's sifaka. Despite the species being sympatric and overlapping dietarily in some specific plant parts, they display considerable ecological, morphological, and behavioral differences as noted above.

### 1.1 Hypotheses

Our null hypothesis (H0) is that food placement on the toothrow is not associated with variation in FMPs, food geometry (shape and size), or species differences related to masticatory morphology. Our alternative hypotheses are not mutually exclusive and may complement each other.

Placement on the toothrow is related to food toughness. We expect that tough foods require higher bite forces to fracture them. In order to fracture a tough food, the lemur has to feed strain energy into a crack to propagate it. The lemur can do this by either biting the food with high force or straining the food at a lower force but over a longer period of time. Given feeding competition and other external pressures, we hypothesize that the lemurs will avoid time‐consuming behaviors if possible and will minimize the time needed to bite into foods to maximize food intake. Therefore, we expect tougher foods regardless of food size and shape or the lemur species to be placed posteriorly on the toothrow to take advantage of the larger forces produced (e.g., Perry et al. 2011a; Ross et al. 2018). Food placement on the toothrow will likely show a stronger correlation with tougher foods than food geometry. In contrast, less tough foods presumably require less force generation to fracture them; therefore, factors such as food size and shape may play a more significant role in determining their placement.

Placement on the toothrow is related to food size. We generally expect larger foods, particularly non‐flat foods, such as fruits, to be placed more anteriorly on the toothrow to take advantage of the potentially wider gape. However, while lemurs may require a wide gape to consume larger foods, there is an inverse relationship between bite force and gape. Therefore, a large or voluminous food that is also tough may be positioned more posteriorly on the toothrow to take advantage of the greater bite force potential (as predicted in H1).

*P. verreauxi* and *L. catta* show interspecific differences in food placement. P. verreauxi and L. catta will position foods differently, depending on the size and toughness of the food. We expect that P. verreauxi will bite tougher foods more anteriorly than L. catta to take advantage of the greater anterior force production resulting from a shorter, more robust jaw (Perry et al. 2011b). While L. catta, with a wider gape, would be able to position larger, bulkier foods more posteriorly to take advantage of higher bite forces posteriorly (e.g., Ross et al. 2018).

## 2 Materials and Methods

This study was approved by the Ethics and Animal Welfare Commission of the University of Veterinary Medicine, Vienna, Austria and adhered to the ASP's ethical principles and best practices for field primatology. It complied with all relevant legal requirements of the host country, Madagascar.

### 2.1 Study Site and Species

The study was conducted at Beza Mahafaly Special Reserve (BMSR; 23°39'25″ S, 44°37'43″E) in southwestern Madagascar. BMSR is a tropical dry forest. Our primary data collection took place in Parcel 1, a semi‐enclosed, 80‐hectare plot with distinct microhabitats, trending from a gallery forest bordering a seasonal river on one side to a dry, deciduous forest (Sussman and Rakotozafy 1994; Axel and Maurer 2010). The lemurs in this study were not exclusively confined to Parcel 1 but ranged outside in the surrounding dry forest/spiny forest areas that exhibited varying degrees of human activities. The region is characterized by distinct wet (October‐April) and dry (May‐August) seasons. During our study period, the site was experiencing an extended, multi‐year drought (Barbosa et al. 2021).

Behavioral data were collected during all‐day focal follows of three groups of *L. catta* and four groups of *P. verreauxi* from May 2019 to August 2019 (dry season) and December 2019 to March 2020 (wet season). The groups overlapped in home ranges and had similar food availability. The species were followed on alternate days 6 days per week. In two seasons, total observation time comprised 842 h in 102 observation days. Focal observations were conducted on the same seven adult individuals per sex (14 total individuals) in the four *P. verreauxi* groups over two seasons. For *L. catta*, seven adult female and two male focal individuals were observed in three groups per season. Focal individuals (*n* = 23) had colored collars and numbered pendants and/or identifying marks. Individuals were followed for approximately 1 h at a time.

### 2.2 Dietary Toughness (FMPs)

Fracture of plant tissues is influenced by their physical and mechanical properties [see Lucas (2004) for further discussion]. We focused on toughness (i.e., resistance to crack propagation), which was measured with an FLS portable tester (Lucas et al. 2001; van Casteren et al. 2016). We tested toughness with cobalt steel scissors (Dovo Solingen) mounted on the testing platform. Plant parts were placed between the scissor blades, and the crosshead of the tester, with an attached load cell and linear variable displacement transducer (LVDT) that measured displacement, was lowered onto them. The resulting force signal was relayed to software that calculated the toughness value for that specimen. An empty pass of the scissors accounted for the friction of the scissor blades and was subtracted from the actual test.

We collected plant parts from branches that were directly adjacent to where the lemurs were observed eating during focal follows. These frequently had bite marks present, which helped guide the selection of parts collected for testing. The plant parts were stored in sealed plastic bags with a little water to prevent dehydration, then brought to the field lab for immediate testing.

Different plant parts (e.g., lamina, petiole) and phenophases (e.g., immature and mature leaves) of the same plant species were tested separately. Toughness data were categorized according to the structural components of plant parts: toughness values from parts with a higher concentration of structural carbohydrates (e.g., rachis, exocarp) were designated as maximum toughness (R_max_), and values from other parts were classified as average toughness (R_av_; e.g., leaf lamina, fruit pulp). As an example of our testing procedure, leaf cuts were made from the outer edge of the leaf toward the midrib. These cuts were focused on the lamina but could also include secondary veins and the midrib. The resulting toughness value represented an average across all of these parts for that leaf (R_av_). If the lemur bit off the leaf at the petiole, then we tested the petiole, which would be classified as a maximum toughness value (R_max_) due to the concentration of structural carbohydrates in this part. The rationale for classifying food parts in this way was to capture potential differences in feeding behaviors related to the material composition of the underlying plant parts. We preferred to assign plant parts into average or maximum toughness categories *prior* to testing to maintain consistency among the tests. Each plant part was tested a minimum of three times, and the values were averaged by plant part.

It is important to note that maximum toughness values are not an absolute number or category. Even though one could expect R_max_ to always be higher than R_av_, in reality, R_max_ in one plant species can be lower than R_av_ in another. These differences reflect inherent biological variation among plant species in the composition and mechanical properties of their tissues. In other words, a particularly tough leaf can be tougher than a structurally carbohydrate‐rich part (R_max_) of another plant species. We generally found that, though there is overlap in these categories, R_max_ is higher than R_av_ in our datasets (Fig. S1; Yamashita et al. 2024). We acknowledge that there are some issues with assigning foods to these categories rather than simply using the highest toughness value as the maximum and lower values as an “average.” A potential issue with this approach is that a threshold for determining what a “maximum” versus an “average” value is would have to be defined. In our approach, the absolute values of R_av_ and R_max_ across plant species are not necessarily directly comparable as discussed above, and some plant parts do not easily fit into either category; e.g., an entire fruit or stalk is bitten in half. However, our toughness categories are based on structural components per plant part that will be unchanging and are not based on threshold values.

### 2.3 Video Analysis

Following the completion of the focal follows, we ranked each lemur species’ most commonly consumed foods (approximately the top ten foods) during each season and analyzed videos for these foods to determine biting placements. Video recordings were made opportunistically during the daily focal follows and included non‐focal individuals. Subsequent analysis of the videos enabled better quantification of food placement on the toothrow. The analysis of the videos was balanced per individual and number of sexes across all groups and major foods. In the current study, 23 individual plant parts and 30 lemur individuals (non‐focal individuals were also included) were analyzed. We used VideoLoupe with a playback speed of 25 frames per second for the analysis (see Table S1 and Flowers et al. 2023 for lists of foods examined).

We define feeding bouts as beginning with the initial introduction of the food to the oral cavity and ending with swallowing. Bites occur during oral preparation up to and including when the food is bitten off. For each feeding bout per plant, we counted the number of bites before chewing and recorded the positions at which these bites were made.

The biting locations were categorized as ANT (food placed at the front of the mouth between the incisors and toothcomb), INT (food placed in the middle of the toothrow intermediate between ANT and POST), and POST (food placed on the posterior part of the postcanines closest to the temporomandibular joint). These categories cannot be assigned to specific tooth positions. Across feeding bouts for the same food, bites were taken at all three bite positions, but generally, one position predominated. We summed the bite numbers for each position for each feeding bout to obtain the total bite numbers for each position for that food part.

### 2.4 Food Geometry Categories

Since the gape is naturally limited by its structure, the shape and size of foods can influence how they are initially placed in the mouth. For example, the lemur species may need a wide gape to consume larger food, bearing in mind that bite force and gape are inversely related but can be partially compensated for morphologically (Eng et al. 2009; Terhune et al. 2011). Foods were categorized as either flat (e.g., leaves; 2D1, 2D2) or non‐flat plant parts (e.g., fruits, flowers, stalks; 3D1, 3D2). The four categories were defined as: 2D1, small leaves < 5 cm; 2D2, medium to large leaves > 5 cm; 3D1, small fruits and flowers < 1.5 cm in the longest dimension; and 3D2, stalks and large fruits > 1.5 cm in the shortest dimension.

### 2.5 Statistical Analysis

This study explores factors influencing where food is first placed in the mouth during oral processing. We evaluated the effects on biting position via multinomial logistic mixed‐effect regression models with an aggregated response variable. This model evaluates a categorical, unordered response variable with multiple potential outcomes. Logistic models estimate probabilities; in our case, the model estimates the probability of a bite location being used with respect to our predictors. All analyses were run in R v. 4.3.1 (R Core Team 2023) with the specific packages described below. We used the R package, mclogit v. 0.9.6 (Elff 2022), to perform a logistic regression with random effects, employing the function mblogit for baseline log‐odds (logit) models.

The response variable, biting position, was categorized into three levels (ANT, INT, POST). The predictors included food toughness (R_av_ or R_max,_ covariates in separate models), food shape and size (2D1, 2D2, 3D1, 3D2), lemur species (*P. verreauxi*, *L. catta*), and season (wet, dry). The videos were analyzed by two observers, and even though we checked for reliability, we also accounted for the observer as an additional effect in the models.

We contrasted models that differed only in their random‐effects structures to determine which random components to include, and chose the model with the lowest Akaike Information Criterion (AIC). In addition to the response variable and predictors above, the final models included lemur individual (*n* = 30) and food species/part (*n* = 23) as random intercepts: mblogit(bite position ~ R_av_ (or R_max_) + species + season + shape + observer, random = c(~1|individual, ~1|food_part)).

We assessed multicollinearity using the variance inflation factor (VIF) function of the car package (Fox and Weisberg 2019), and standardized the covariates (R_av_ and R_max_) as *Z*‐scores after finding VIF values higher than 7.0 for some predictors. The resulting VIF values were below 2.0 for both R_av_ and R_max_ models.

We first obtained the log odds (coefficients from the models) and odds ratios from the logistic regressions. Then, we conducted posthoc pairwise contrasts with the emmeans (estimated marginal means) R package v.1.8.7 (Laird et al. 2023). The emmeans package takes the fitted model (ensuring that the statistical parameters and original model remain unchanged) and applies post‐hoc tests. Emmeans allows for the simultaneous assessment of contrasts and their significance levels. We accounted for multiple testing with the Bonferroni‐Holm method. The emmip function of emmeans and the ggplot2 package (Wickham 2016) were used for visualization of the results.

The dataset used for the analysis has been provided in Table S1.

## 3 Results

### 3.1 Toughness

**Figure 1:**
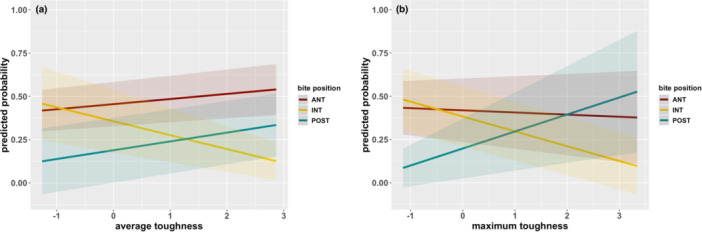
Contrasts of predicted bite positions for models with (a) average toughness, R_av_, and (b) maximum toughness, R_max_, as the covariate. Slopes are estimated for toughness at each bite position using the emmeans package. Plots were made with the emmeans function, emmip, then customized in ggplot2. See Tables 1, 2 for results and significance levels of pairwise contrasts between bite positions and tests to determine if the slopes differ significantly from zero at each bite position. R_av_ and R_max_ have been *Z*‐scaled. Confidence intervals are 95%. ANT, anterior of mouth; INT, intermediate toothrow; POST, posterior toothrow. See text for further details.

The probabilities of using ANT and POST do not change with average toughness (R_av_), while INT usage decreases with increasing average toughness (Figure 1a; Table 1). With respect to R_max_, the probability of using POST increases significantly as R_max_ increases; the probabilities of using ANT and INT are not correlated with maximum toughness (Figure 1b; Table 2).

**Table 1:** (a) Comparisons of R_av_ slope differences between bite positions and (b) if the slopes themselves differ significantly from zero at each bite position.

Bite position contrast	Estimate	SE	*Z*‐ratio	*p*‐value	Adjusted *p*‐value
**(a)**					
ANT ‐ INT	0.5716	0.2341	2.442	0.0146	**0.0292**
ANT ‐ POST	−0.2231	0.2992	−0.746	0.4558	0.4558
INT ‐ POST	−0.7947	0.2511	−3.165	0.0016	**0.0047**
**(b)**					
ANT	0.1271	0.1529	0.831	0.4057	0.4057
INT	−0.4445	0.1464	−3.035	0.0024	**0.0072**
POST	0.3502	0.1699	2.062	0.0392	0.0784

*Note:* Slopes are estimated for toughness at each bite position with the emmeans package. Results are given on the log‐odds ratio scale. *p*‐value adjustment by the Bonferroni‐Holm method. Significant results (*p* < 0.05) are in bold.

Abbreviations: ANT, anterior of mouth; INT, intermediate position of postcanine toothrow; POST, posterior‐most part of toothrow; R_av_, average toughness.

**Table 2:** (a) Comparisons of R_max_ slope differences between bite positions and (b) if the slopes themselves differ significantly from zero at each bite position.

Bite position contrast	Estimate	SE	*Z*‐ratio	*p*‐value	Adjusted *p*‐value
**(a)**					
ANT ‐ INT	0.3231	0.3362	0.961	0.3364	0.3364
ANT ‐ POST	−0.6722	0.4358	1.542	0.1230	0.2459
INT ‐ POST	−0.9954	0.3517	−2.830	0.0047	**0.0140**
**(b)**					
ANT	−0.1422	0.2438	0.244	0.5597	0.5597
INT	−0.4653	0.2158	−2.156	0.0311	0.0622
POST	0.5300	0.2091	2.535	0.0112	**0.0337**

*Note:* Slopes are estimated for toughness at each bite position with the emmeans package. Results are given on the log odds ratio scale. *P*‐value adjustment by the Bonferroni‐Holm method. Significant results (*p* < 0.05) are in bold.

Abbreviations: ANT, anterior of mouth; INT, intermediate position of postcanine toothrow; POST, posterior‐most part of toothrow; R_max_, maximum toughness.

### 3.2 Food Geometry

**Figure 2:**
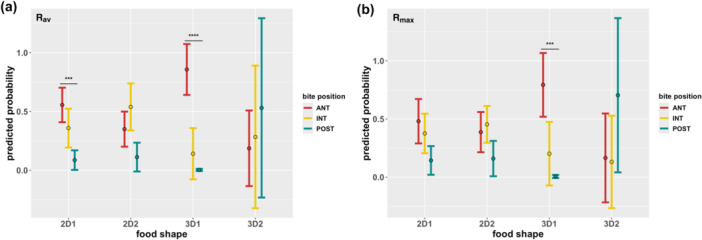
Probability of bite position use by food shape with R_av_ and R_max_ as a covariate. Estimated marginal means are derived from the results of the multinomial logistic regression model and are used to derive the predicted response. Probabilities for each bite position by shape are depicted by the center circle, and colored whiskers are 95% confidence intervals. Plots were made with the emmeans function, emmip, then customized in ggplot2. See Table 3 for results and significance levels of each pairwise contrast of bite position within shapes. R_av_ and R_max_ have been *Z*‐scaled. ANT, anterior of mouth; INT, intermediate toothrow; POST, posterior toothrow; 2D1, 2D2 = flat shapes; 3D1, 3D2 = non‐flat shapes. See text for further details. ***p < 0.001, ****p < 0.0001.

Flat foods that are 2D1 and 2D2 are more likely to be processed ANT and INT in both R_av_ and R_max_ models, though the contrasts are not always significant. 3D1 shapes are less likely to be processed INT and POST, indicating that they are most likely to be processed ANT in both models. 3D2 shapes are more likely to be processed POST in both models (Figure 2; Table 3).

**Table 3:** Pairwise contrasts (emmeans) of bite position by food geometry in (a) R_av_ model and (b) R_max_ model.

Shape	Bite position contrast	Estimate	SE	*Z*‐ratio	*p*‐value	Adjusted *p*‐value
**(a) R_av_ model**						
2D1	ANT ‐ INT	0.8011	0.6461	1.240	0.2150	1.0000
	ANT ‐ POST	2.5790	0.6303	4.092	< 0.0001	**0.0005**
	INT ‐ POST	1.7779	0.7757	2.292	0.0219	0.1971
2D2	ANT ‐ INT	−0.7756	0.7062	−1.098	0.2721	1.0000
	ANT ‐ POST	1.4484	0.6877	2.106	0.0352	0.2816
	INT ‐ POST	2.2240	0.9509	2.339	0.0194	0.1935
3D1	ANT ‐ INT	3.5946	1.8148	1.981	0.0476	0.2858
	ANT ‐ POST	7.5674	1.6306	4.641	< 0.0001	**< 0.0001**
	INT ‐ POST	3.9728	1.9051	2.085	0.0370	0.2816
3D2	ANT ‐ INT	−0.5435	1.5982	−0.340	0.7338	1.0000
	ANT ‐ POST	−1.5933	2.4039	−0.663	0.5075	1.0000
	INT ‐ POST	−1.0497	3.0169	−0.348	0.7279	1.0000
**(b) R_max_ model**						
2D1	ANT ‐ INT	0.4306	0.7154	0.602	0.5473	1.0000
	ANT ‐ POST	1.7096	0.7782	2.197	0.0280	0.2803
	INT ‐ POST	1.2791	0.6830	1.873	0.0611	0.4889
2D2	ANT ‐ INT	−0.2734	0.6197	−0.441	0.6591	1.0000
	ANT ‐ POST	1.2004	0.8368	1.434	0.1514	0.9087
	INT ‐ POST	1.4738	0.7622	1.934	0.0532	0.4785
3D1	ANT ‐ INT	2.7206	1.7181	1.583	0.1133	0.7932
	ANT ‐ POST	6.4982	1.5080	4.309	< 0.0001	**0.0002**
	INT ‐ POST	3.7776	1.4463	2.612	0.0090	0.0990
3D2	ANT ‐ INT	0.2734	1.7123	0.160	0.8731	1.0000
	ANT ‐ POST	−2.4864	2.9163	−0.853	0.3939	1.0000
	INT ‐ POST	−2.7598	3.2823	−0.841	0.4005	1.0000

*Note:* Results are given on the log‐odds ratio scale. *p*‐value adjustment by the Bonferroni‐Holm method. Significant results (*p* < 0.05) are in bold. See text for description of abbreviations for food geometry.

Abbreviations: ANT, anterior of mouth; INT, intermediate position of postcanine toothrow; POST, posterior‐most part of toothrow; R_av_, average toughness; R_max_, maximum toughness.

### 3.3 Lemur Species

**Figure 3:**
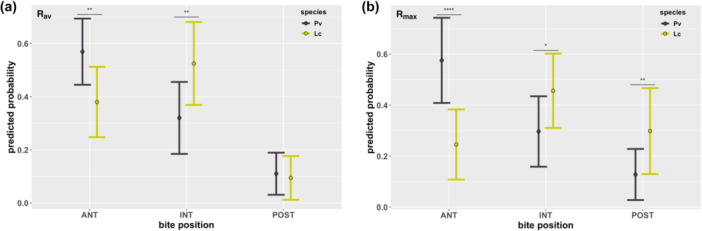
Probability of bite position use by lemur species with R_av_ and R_max_ as a covariate. Estimated marginal means are derived from the results of the multinomial logistic regression model. Probabilities for each bite position by species are depicted by the center circle, and colored whiskers are 95% confidence intervals. Plots were made with the emmeans function, emmip, then customized in ggplot2. See Table 4 for results and significance levels of each pairwise contrast of bite position between and within species. R_av_ and R_max_ have been *Z*‐scaled. ANT, anterior of mouth; Lc, *Lemur catta*; INT, intermediate toothrow; POST, posterior toothrow; Pv, *Propithecus verreauxi*. See text for further details. *p < 0.05, **p < 0.01, ****p < 0.0001.

The two lemur species show differences in initial biting positions in both R_av_ and R_max_ models, with *P. verreauxi* more likely to bite foods ANT than *L. catta* and *L. catta* more likely to bite INT than *P. verreauxi* (Figure 3; Table 4). *L. catta* is significantly more likely than *P. verreauxi* to use POST in the R_max_ model; there are no differences between species in POST in the R_av_ model. Within species, *P. verreauxi* is more likely to use ANT than POST. The other positions do not differ in the likelihood of use. *L. catta* is not likely to favor one biting position over another in the R_max_ model, but POST usage is significantly less likely than either ANT or INT in the R_av_ model.

**Table 4:** Pairwise contrasts (emmeans) of bite position by species in (a) R_av_ model and (b) R_max_ model.

	Contrast	Estimate	SE	*Z*‐ratio	*p*‐value	Adjusted *p*‐value
**(a) R_av_ model**						
Contrast by bite position					
ANT	*Pv ‐ Lc*	0.7694	0.2655	2.898	0.0038	**0.0075**
INT	*Pv ‐ Lc*	−0.8538	0.2762	−3.092	0.0020	**0.0060**
POST	*Pv ‐ Lc*	0.1690	0.4226	0.400	0.6893	0.6893
Contrast within species					
*Pv*	ANT ‐ INT	1.0330	0.5499	1.878	0.0603	0.1206
	ANT ‐ POST	2.3690	0.5241	4.520	< 0.0001	**< 0.0001**
	INT ‐ POST	1.3360	0.6178	2.163	0.0306	0.0917
*Lc*	ANT ‐ INT	−0.5902	0.5825	−1.013	0.3111	0.3111
	ANT ‐ POST	1.7686	0.5672	3.118	0.0018	**0.0073**
	INT ‐ POST	2.3588	0.7128	3.309	0.0009	**0.0047**
**(b) R_max_ model**						
Contrast by bite position						
ANT	*Pv ‐ Lc*	1.4268	0.3400	4.197	< 0.0001	**< 0.0001**
INT	*Pv ‐ Lc*	−0.6884	0.3140	−2.193	0.0283	**0.0283**
POST	*Pv ‐ Lc*	−1.0639	0.3621	−2.938	0.0033	**0.0066**
Contrast within species						
*Pv*	ANT ‐ INT	1.1685	0.6520	1.792	0.0731	0.3470
	ANT ‐ POST	2.2253	0.7159	3.108	0.0019	**0.0113**
	INT ‐ POST	1.0568	0.5820	1.816	0.0694	0.3470
*Lc*	ANT ‐ INT	−0.9468	0.5476	−1.729	0.0838	0.3470
	ANT ‐ POST	−0.2655	0.6983	−0.380	0.7038	0.7038
	INT ‐ POST	0.6813	0.6430	1.060	0.2894	0.5787

*Note:* Results are given on the log‐odds ratio scale. *p*‐value adjustment by the Bonferroni‐Holm method. Significant results (*p* < 0.05) are in bold.

Abbreviations: ANT, anterior of mouth; INT, intermediate position of postcanine toothrow; *Lc*, *Lemur catta*; POST, posterior‐most part of toothrow; *Propithecus verreauxi*; R_av_, average toughness; R_max_, maximum toughness.

### 3.4 Season

**Figure 4:**
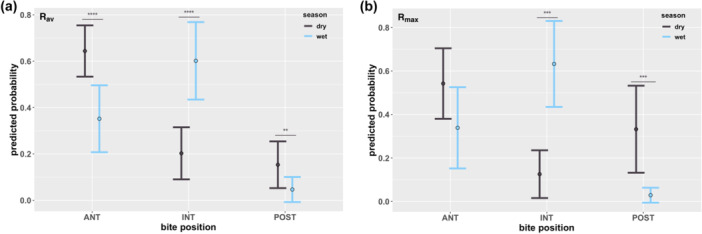
Probability of bite position use with respect to season with R_av_ (a) and R_max_ (b) as a covariate. Estimated marginal means are derived from the results of the multinomial logistic regression model. Probabilities for each bite position by season are depicted by the center circle, and colored whiskers are 95% confidence intervals. Plots were made with the emmeans function, emmip, then customized in ggplot2. See Table 5 for results and significance levels of each pairwise contrast of bite position between and within seasons. R_av_ and R_max_ have been *Z*‐scaled. ANT, anterior of mouth; D, dry season; INT, intermediate toothrow; POST, posterior toothrow; W, wet season. See text for further details. **p < 0.01, ***p < 0.001, ****p < 0.0001.

We model potential seasonal differences in biting location as an indicator of the underlying differences in food composition in the wet and dry seasons (Yamashita et al. 2024). When comparing how biting locations differ seasonally, in the R_av_ model (Figure 4; Table 5a,b), ANT is significantly more likely to be used during the dry season. INT and POST use are more likely in the wet and dry seasons, respectively, in both R_av_ and R_max_ models (Figure 4; Table 5).

**Table 5:** Pairwise contrasts (emmeans) of bite position by season in (a) R_av_ model and (b) R_max_ model.

	Contrast	Estimate	SE	*Z*‐ratio	*p*‐value	Adjusted *p*‐value
**(a) R_av_ model**						
Contrast by bite position						
ANT	dry‐wet	1.2035	0.2546	4.728	< 0.0001	**< 0.0001**
INT	dry‐wet	−1.7815	0.2348	−7.587	< 0.0001	**< 0.0001**
POST	dry‐wet	1.3096	0.5021	2.608	0.0091	**0.0091**
Contrast within season						
Dry	ANT ‐ INT	1.9618	0.5380	3.646	0.0003	**0.0008**
	ANT ‐ POST	2.3005	0.5499	4.184	< 0.0001	**0.0002**
	INT ‐ POST	0.3388	0.6431	0.527	0.5984	0.5984
Wet	ANT ‐ INT	−1.0232	0.6700	−1.527	0.1267	0.2535
	ANT ‐ POST	2.4066	0.6166	3.903	0.0001	**0.0004**
	INT ‐ POST	3.4298	0.8691	3.946	0.0001	**0.0004**
**(b) R_max_ model**						
Contrast by bite position						
ANT	dry‐wet	0.8387	0.4553	1.842	0.0655	0.0655
INT	dry‐wet	2.4847	0.5489	−4.527	< 0.0001	**< 0.0001**
POST	dry‐wet	2.8206	0.6224	4.532	< 0.0001	**< 0.0001**
Contrast within season						
Dry	ANT ‐ INT	2.1116	0.5971	3.536	0.0004	**0.0016**
	ANT ‐ POST	0.8680	0.7614	1.140	0.2543	0.4522
	INT ‐ POST	−1.2436	0.8655	−1.437	0.1507	0.4522
Wet	ANT ‐ INT	−1.2119	0.8563	−1.415	0.1570	0.4522
	ANT ‐ POST	2.8498	0.6665	4.276	< 0.0001	**< 0.0001**
	INT ‐ POST	4.0617	0.8915	4.556	< 0.0001	**< 0.0001**

*Note:* Results are given on the log odds ratio scale. *p*‐value adjustment by the Bonferroni‐Holm method. Significant results (*p* < 0.05) are in bold.

Abbreviations: ANT, anterior of mouth; INT, intermediate position of postcanine toothrow; POST, posterior‐most part of toothrow; R_av_, average toughness; R_max_, maximum toughness; dry, dry season; wet, wet season.

Within seasons, the lemurs are significantly more likely to use ANT than the other positions in the dry season. In both models, ANT was more likely to be used than INT in the dry season, and POST usage was the least likely in the wet season.

Forest plots of odds ratios of the likelihood of bite location use with respect to food shape/size, lemur species, and season are in Figures S2–4.

## 4 Discussion

Contrary to the null hypothesis, we found that food toughness, lemur species, and some food geometries influence initial food placement on the toothrow. In addition, due to seasonal availability and consumption of specific food plants, the bite positions that are used with those foods result in seasonal differences in biting positions.

### 4.1 Food Placement and Toughness

As expected in our first hypothesis (H1), we found that food placement is related to the toughness of the food. Both R_max_ and R_av_ are correlated with positioning the food further back on the toothrow (in the POST position) to likely take advantage of the greater force generation closer to the temporomandibular joint (TMJ). Similar results were found for *Cercocebus*, which relies on postcanines to access tough foods (Daegling and McGraw 2007; McGraw et al. 2011). However, toughness alone does not determine food placement in our study, as found in Yamashita (2003) and for *Sapajus libidinosus* (Laird et al. 2020), suggesting that multiple factors, in addition to toughness, influence this process. These findings suggest that food placement reflects an integrated response to both the mechanical demands of the food and the functional capabilities of the masticatory apparatus, rather than toughness in isolation.

### 4.2 Food Placement and Geometry

We initially expected that the larger food items would be positioned more anteriorly to take advantage of the wider gape at the front of the mouth (H2). Interestingly, our results indicate that the strongest relationship between initial food placement and geometry was the higher likelihood of using ANT for smaller shapes (3D1), such as small fruits and flowers, and ANT for small leaves (2D1) in both R_av_ and R_max_ models (Figure 2, S5; Table 3). Both lemur species quickly ate small foods at the front of the mouth, so, in addition to some potential gape limitations for large foods (see below), plucking small foods anteriorly with help from the tongue against the hard palate may be more efficient for rapid food intake.

The effects of food geometry on ingestion are also modulated by interactions with food toughness and species morphology (Figure S5). For example, when comparing lemur species, we found that *P. verreauxi* consistently used ANT more than *L. catta* (Figures 3, S5; Table 4). *P. verreauxi*'s masticatory apparatus emphasizes greater robusticity with a relatively shorter jaw (e.g., Groves and Helgen 2007; Perry et al. 2011b) compared to that of *L. catta* (Diogo et al. 2014), which may limit gape. In terms of relative food size, what may be a large food part for *P. verreauxi* may not be for *L. catta*. In support of this, *L. catta* is more likely to use more posterior positions than *P. verreauxi* across all food geometries examined (Figure S5).

### 4.3 Food Placement by Season

The lemurs consumed different food species seasonally or had a higher consumption of a particular food in one season, which contributed to the seasonal preferences found in biting location (Figure 4, Table 5, Table S1). Differences in ANT bites, where the dry season usage was higher than the wet, mostly resulted from higher consumption of *Metaporana parvifolia* (kililo) leaves by both lemur species, which tend to bite this plant anteriorly. Other foods that contributed to higher dry season ANT placement were mostly eaten by *P. verreauxi* (e.g., old *Tamarindus indica* [kily] fruit, *Acacia bellula* [tratriotse] young leaves, *Grewia* sp. [maintyfototse] young leaves). The lemurs were more likely to use INT in the wet season, driven primarily by *L. catta*'s consumption of *Cedrelopsis grevei* (katrafay) young leaves and secondarily by consumption of *Acacia rovumae* (robontse) young leaves by *P. verreauxi* in this season. The higher likelihood of dry season POST usage (in both models) was led by feeding on old *Tamarindus* fruit by *L. catta* and *Euphorbia tirucalli* (famata) stalks by *P. verreauxi*. The seasonal differences in ANT and INT usage reflect species‐specific preferences for food placement (Figure S5) and are linked to greater consumption of particular plants by each lemur species. While season may not directly alter feeding behavior, it influences food availability and consumption patterns, which in turn affect the frequency of certain behaviors and the resulting stress patterns in the masticatory apparatus. Because these variations recur seasonally, they can then have cumulative long‐term impacts on cranio‐dental morphology.

### 4.4 Food Placement and Lemur Species

Initial placement of foods during feeding appears to be less of a priority for *L. catta* than for *P. verreauxi*. *P. verreauxi* prioritizes anterior biting in contrast to posterior in both toughness models, and *L. catta* either does not prioritize (for R_max_) or shows significance when not using the posterior position (R_av_) (Figure 3, Table 4). Two primary evolutionary strategies for coping with feeding constraints are morphological adaptations and behavioral responses. Behavioral flexibility, or plasticity (see Strier 2017), refers to the capacity to modify behavior in response to specific environmental challenges (Amici et al. 2018). While complex behaviors such as tool use often come to mind, simpler actions, such as behavioral flexibility in selecting specific initial biting positions, can also be adopted. *P. verreauxi* exhibits anatomical features that optimize food processing, while *L. catta* shows greater flexibility in feeding behaviors (Flowers et al. 2023).

*P. verreauxi* possesses a combination of stronger adductor muscles and a more robust and shorter jaw than *L. catta* (e.g., Perry et al. 2011b), which may contribute to generating higher anterior bite forces. In addition, *P. verreauxi* has a partially fused mandibular symphysis, whereas that of *L. catta* is unfused and presumably weaker since fusion strengthens the symphysis, allowing it to better withstand the increased wishboning that occurs during forceful unilateral mastication (Hylander et al. 2000). Partial fusion appears to be sufficient for *P. verreauxi* to resist such stresses and strains due to the anterior inclination and elongation of the symphysis (Knigge et al. 2020). As a folivore, *P. verreauxi* is predicted to have a smaller gape than *L. catta* (Perry et al. 2011b), which coincides with more robust jaw characteristics and a diet consisting of tougher foods (e.g., Flowers et al. 2023; Yamashita 2002). Additionally, the toothcomb—a specialized dental structure in strepsirrhines comprised of the lower incisors and canines—is more robust in *P. verreauxi* than in *L. catta*, consisting of four stout teeth compared to six slender ones, respectively (e.g., Martin 1972; Schwartz 1974). In extant taxa, the toothcomb serves in both grooming and feeding functions (e.g., Cuozzo and Yamashita 2006; Martin 1972; Sauther et al. 2002).

These features elucidate why *P. verreauxi* uses anterior biting (ANT) more than *L. catta* (Figure 3, Table 4). With its smaller gape (Perry et al. 2011b), *P. verreauxi* cannot position non‐flat foods as far back in the toothrow as *L. catta*. However, it does ingest tougher foods posteriorly, but at a higher toughness threshold than *L. catta*. Another primate known for its masticatory robusticity, *Sapajus libidinosus*, also typically relies more on its anterior teeth during feeding (Laird et al. 2020). Despite the phylogenetic distance between these two primates, this indicates a connection between relative masticatory robusticity and feeding behavior, which may help infer feeding behaviors in fossil species.

In contrast, *L. catta* bites more on the middle of the toothrow (INT) (Figure 3, Table 4), which aligns with its lower anterior bite force potential given its morphology. For example, the less pronounced zygomatic arch in *L. catta* provides a smaller area for adductor muscle attachment, and an unfused mandibular symphysis limits resistance to high anterior stress (Hylander et al. 2000). Perry et al. (2011b) suggest that a wide gape is vital for frugivores, but not necessarily the ability to generate high forces at such gape, since fruits can be large but not particularly mechanically challenging.

Both species use posterior biting (POST) similarly to process non‐flat and tough foods (Figures S8–9). In the case of foods such as *Tamarindus* fruit and *Euphorbia* stalks (3D2 foods), bite placement is influenced by their shape as well as the force required to bite them. Though gape is limited more posteriorly, bite force will be greater near the TMJ. Ripe *Tamarindus* fruit was the largest food eaten (average minimum width = 13.9 mm, maximum width = 25.1 mm, and length = 90.6 mm; *n* = 30; see also Yamashita 2008; Yamashita et al. 2012). However, during the period of this study, *P. verreauxi* and *L. catta* primarily consumed old tamarind fruit (presumably through drought‐related lack of ripe fruit), which was often in pieces or held together by the internal threads. The lemur species ingested it differently, with *P. verreauxi* using more ANT while *L. catta* alternated between INT and POST. The old *Tamarindus* fruit was not the largest food eaten, but it was still mechanically challenging. In this case, the shape and size of the fruit was not as much of a factor as bite force. *L. catta* positioned *Tamarindus* more posteriorly on the toothrow. Slender but tough cylindrical *Euphorbia* stalks (3D2), eaten by *P. verreauxi*, were similarly placed on the postcanines for initial biting.

In summary, *L. catta* uses the INT biting position when feeding on presumably the toughest foods where it can generate larger bite forces, and *P. verreauxi* initially relies on ANT as a default, consistent with its robust morphology, but shifts to more posterior tooth locations as foods become tougher and exceed its anterior processing threshold (Figure S5).

Our findings build on previous work by Yamashita (2003) on the same population, which highlighted the complex relationship between food properties and feeding behavior. That study found that *L. catta* and *P. verreauxi* ingested tougher food using their postcanine teeth. However, when analyzing leaves and fruits separately, factors such as food size and shape became a significant predictor of initial placement in the mouth, with larger leaves and bulkier fruits or stalks more likely to be ingested posteriorly. Unlike in Yamashita's earlier finding, *P. verreauxi* in our study used ANT more frequently. This difference may reflect underlying differences in resource availability (possibly related to the multi‐year drought in the present study), highlighting how ecological variation can guide feeding behavior. Our results highlight the importance of long‐term data collection to capture environmental fluctuations that may even include climate change.

The observed relationship between morphological potential (e.g., stronger adductor muscles, a more robust and shorter jaw, and a partially fused symphysis) and feeding behavior (use of the toothrow) underscores the specialization of *P. verreauxi* for powerful anterior processing. Conversely, *L. catta* adjusts its feeding behaviors more when consuming tougher foods (Flowers et al. 2023) and demonstrates a greater degree of behavioral flexibility. This aligns with *L. catta* being a more generalist species than *P. verreauxi* in terms of diet (e.g., Yamashita 2002) that inhabits a wider range of environments and has a broader geographic distribution (Goodman et al. 2006). Its morphology and behavior should reflect this versatility, as pointed out by Cuozzo and Yamashita (2006), who expected a weaker relationship between tooth morphology and diet for *L. catta* compared to *P. verreauxi*.

We acknowledge that separating food geometry from toughness is quite challenging because they often co‐vary. The way food fractures during a bite is influenced by its shape. Although our models take geometry into account, the non‐homogeneous structure of foods is difficult to fully characterize (Lucas 2004).

Initial food placement on the toothrow affects the stress and strain experienced by the craniodental apparatus. Understanding bite force distribution, jaw mechanics, and how primates maximize efficiency in biting can provide clues to the evolution of primate craniofacial morphology.

## Author Contributions

**Mariana Dutra Fogaça:** conceptualization, methodology, investigation, supervision, visualization, writing – original draft. **Nina Flowers:** conceptualization, investigation, writing – review and editing. **Haja Fabrice Razafindrabe Maminiaina:** investigation, writing – review and editing. **Jean Claude Razafimampiandra:** investigation, writing – review and editing. **Nayuta Yamashita:** conceptualization, data curation, formal analysis, funding acquisition, investigation, methodology, project administration, resources, supervision, validation, writing – review and editing.

## Conflicts of Interest

The authors declare no conflicts of interest.

## Supporting information


Supporting File 1

Supporting File 2

## Data Availability

The data supporting the findings reported here are provided in Table S1.
