# Supplementary material for: How Food Geometry and Toughness Influence Ingestive Patterns in Lemurs
Source: Am J Primatol. 2026 Jul 12;88(7):e70188. doi: 10.1002/ajp.70188 (PMC13358327; doi:10.1002/ajp.70188)
Supplement: Supplementary file 2 — Supporting File 2 [file AJP-88-e70188-s001.pdf]

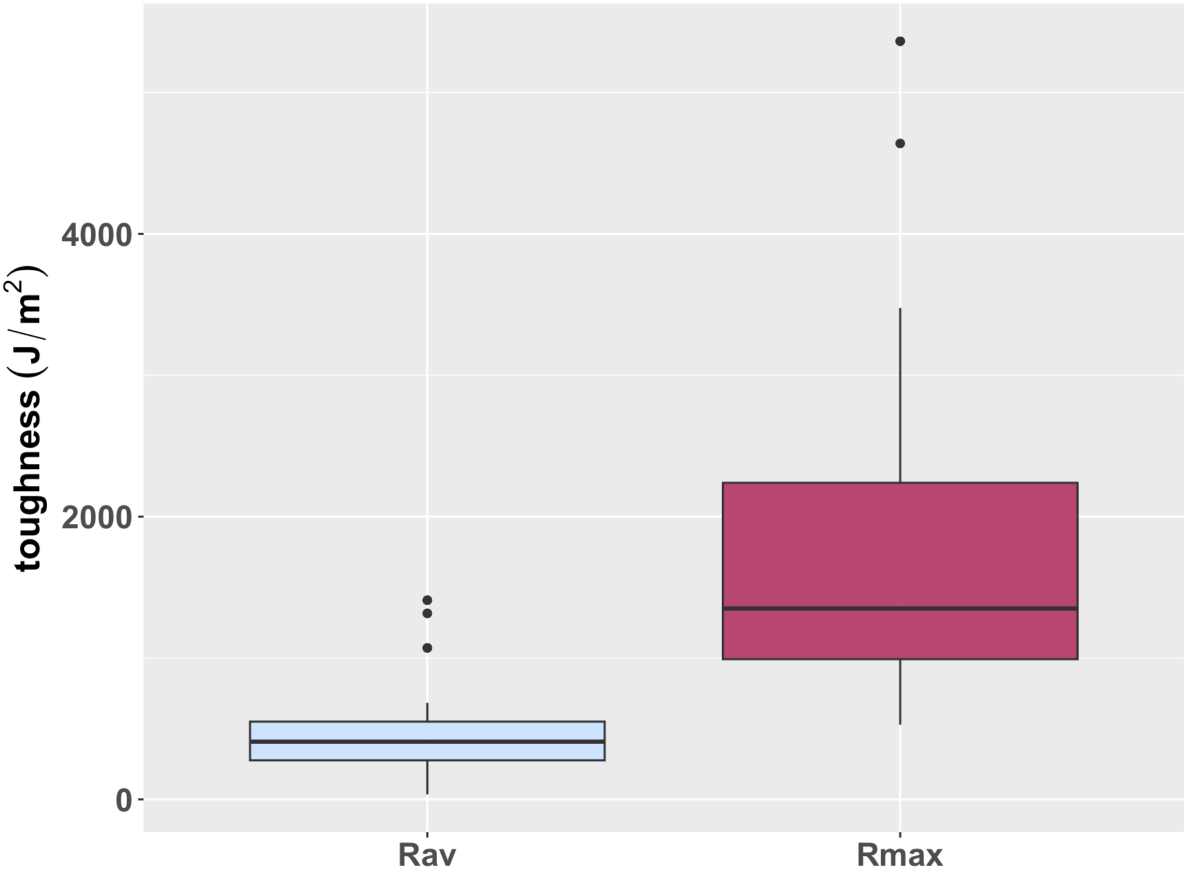

FIGURE S1: Comparison of average and maximum toughness values. Values are untransformed. Boxes represent the central half of the data with the median shown as a line, whiskers extend 1.5 times the interquartile range from the outer margins of the box, and outliers are datapoints beyond this limit. Plots were made in ggplot2. Rav, average toughness; Rmax, maximum toughness. See text for further details on toughness values.

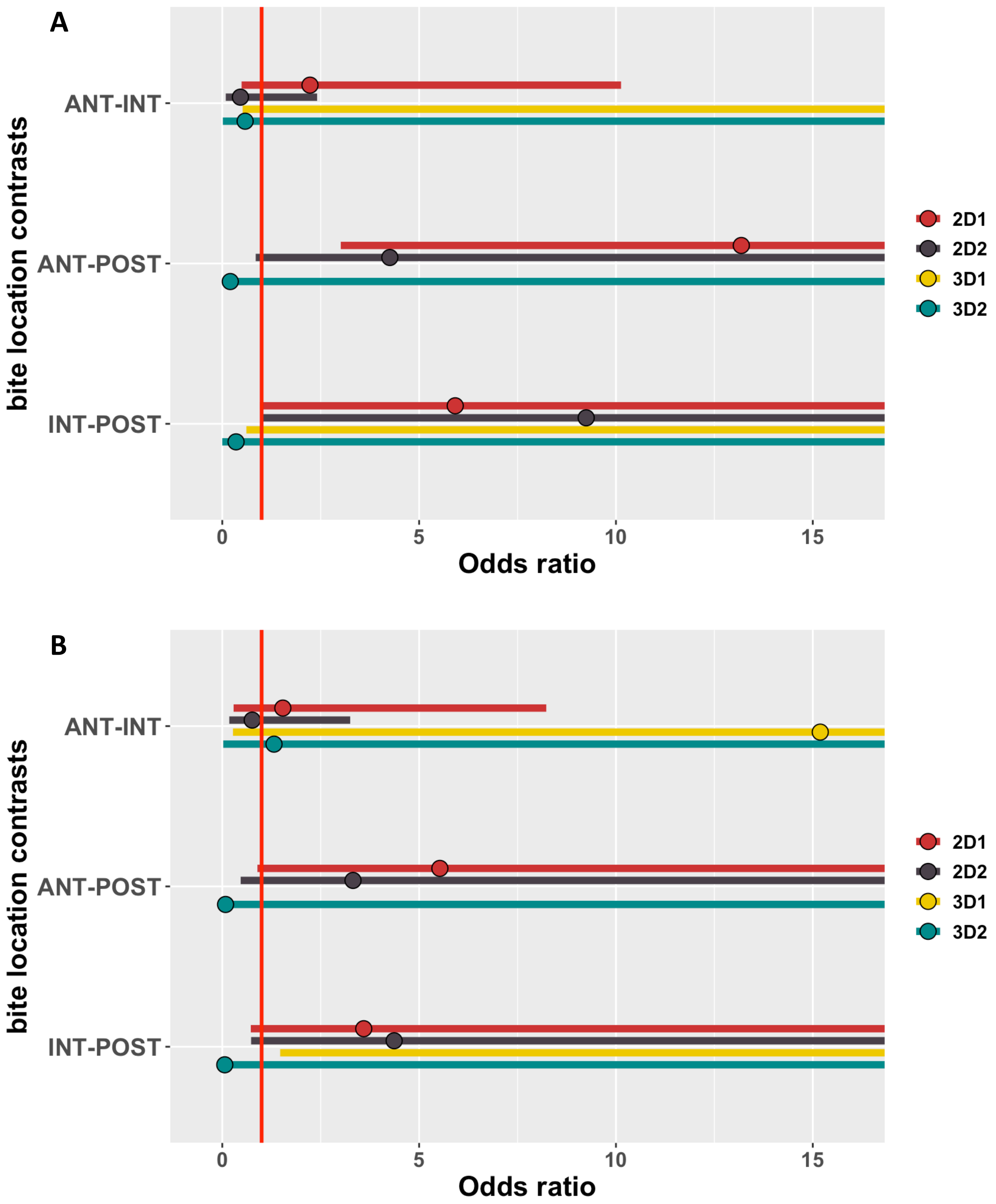

FIGURE S2: Forest plots of odds ratio of contrasts of likelihood of bite location use with respect to food geometry with A) R_{av} and B) R_{max} as a covariate. Plots were made in ggplot2 with values derived from emmeans. Estimated marginal means are estimated from the results of the multinomial logistic regression models, in which R_{av} and R_{max} have been Z-scaled. Contrasts are presented on the log-odds ratio scale. The solid circle represents the odds ratio with 95% confidence intervals. The contrast is significant if the CIs do not cross the vertical red line. The X-axis has been truncated for clarity. ANT, anterior of mouth; INT, intermediate position of postcanine toothrow; POST, posterior-most part of toothrow; 2D1, 2D2=flat geometries; 3D1, 3D2=non-flat geometries.

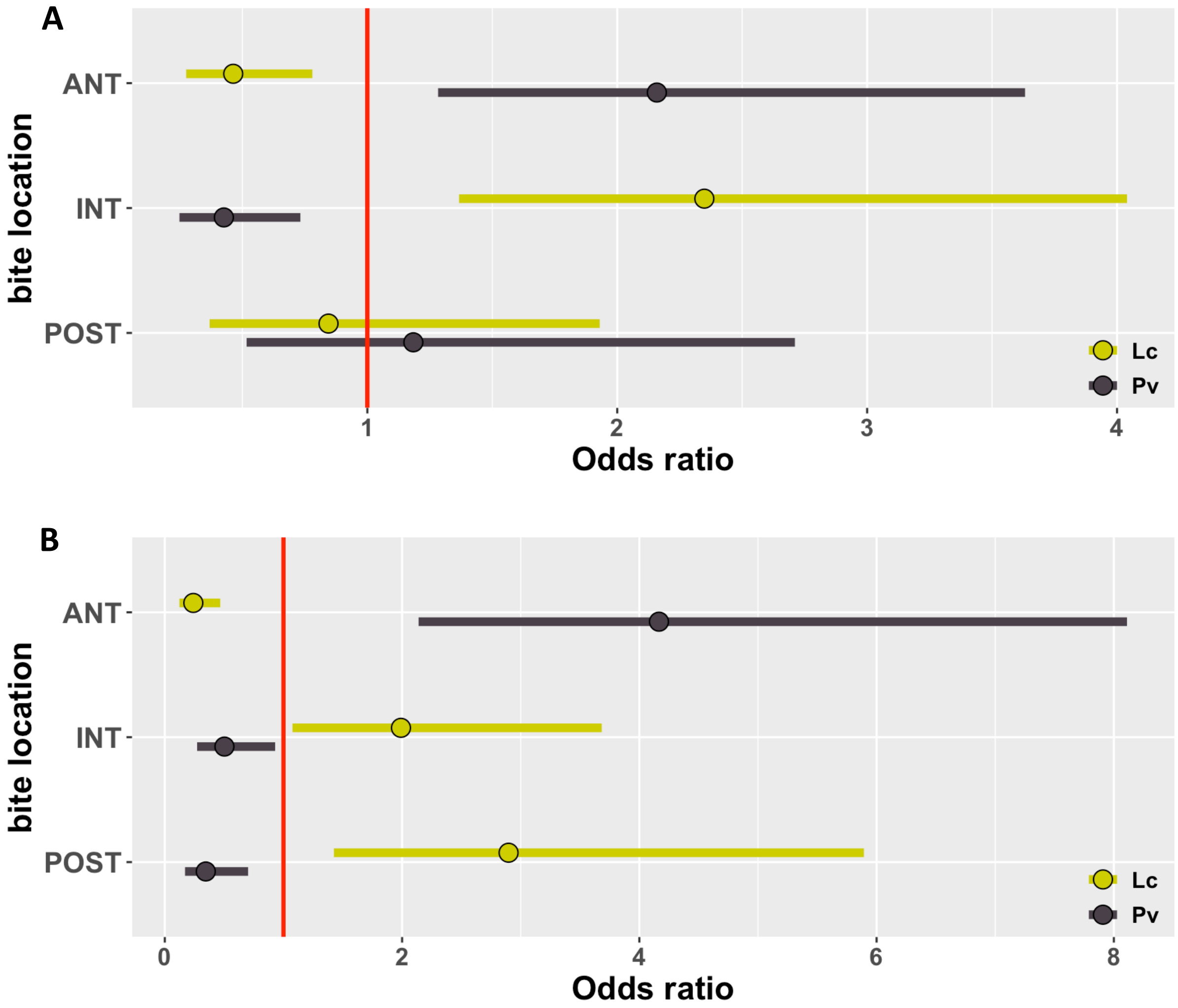

FIGURE S3: Forest plots of odds ratio of likelihood of bite location use by lemur species with A) R_{av} and B) R_{max} as a covariate. Plots were made in ggplot2 with values derived from emmeans. Estimated marginal means are estimated from the results of the multinomial logistic regression model, in which R_{av} and R_{max} have been Z-scaled. Contrasts are presented on the log-odds ratio scale. The solid circle represents the odds ratio with 95% confidence intervals. The contrast is significant if the CIs do not cross the vertical red line. ANT, anterior of mouth; INT, intermediate position of postcanine toothrow; POST, posterior-most part of toothrow; Pv, *Propithecus verreauxi*; Lc, *Lemur catta*.

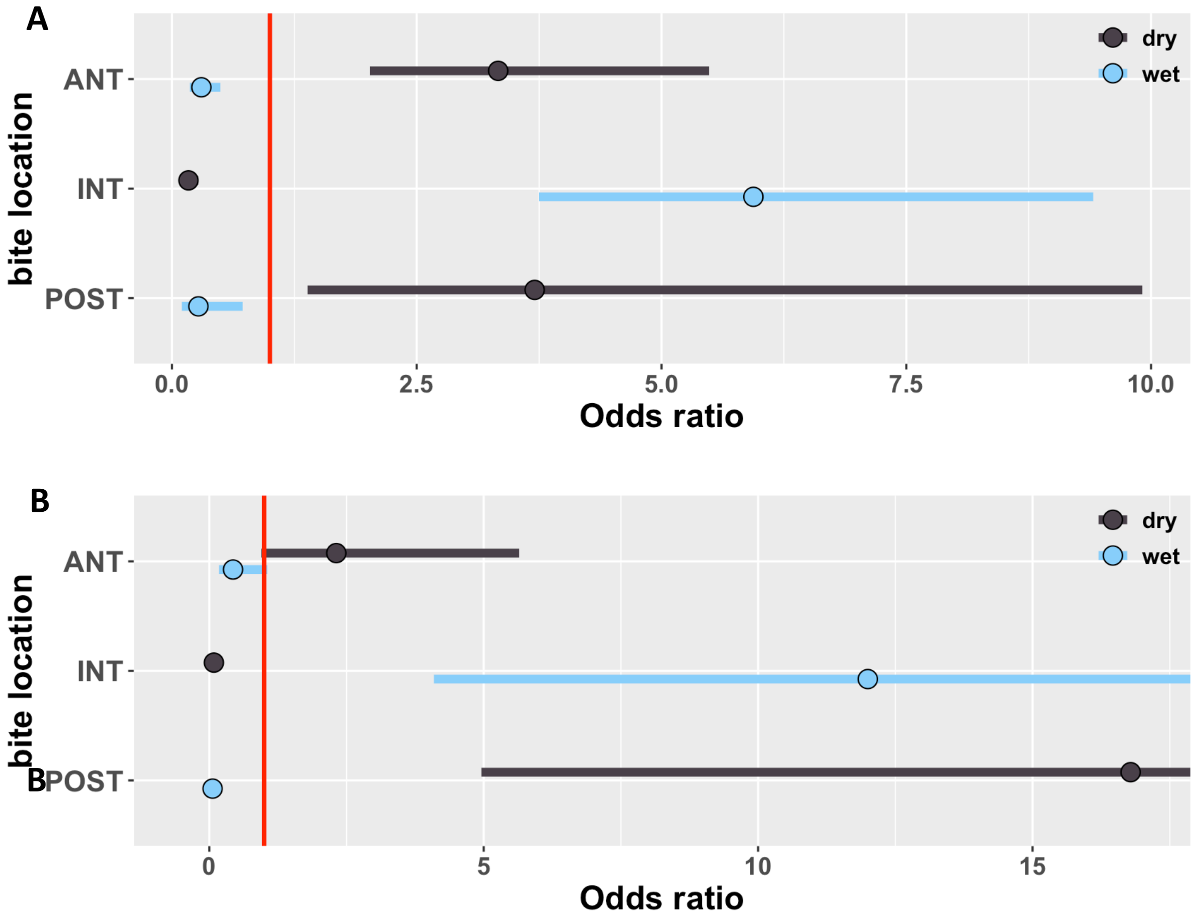

FIGURE S4: Forest plots of odds ratio of likelihood of bite location use in different seasons with A) R_{av} and B) R_{max} as a covariate. Plots were made in ggplot2 with values derived from emmeans. Estimated marginal means are estimated from the results of the multinomial logistic regression model, in which R_{av} and R_{max} have been Z-scaled. Contrasts are presented on the log-odds ratio scale. The solid circle represents the odds ratio with 95% confidence intervals. The contrast is significant if the CIs do not cross the vertical red line. ANT, anterior of mouth; INT, intermediate position of postcanine toothrow; POST, posterior-most part of toothrow; dry, dry season; wet, wet season.

A

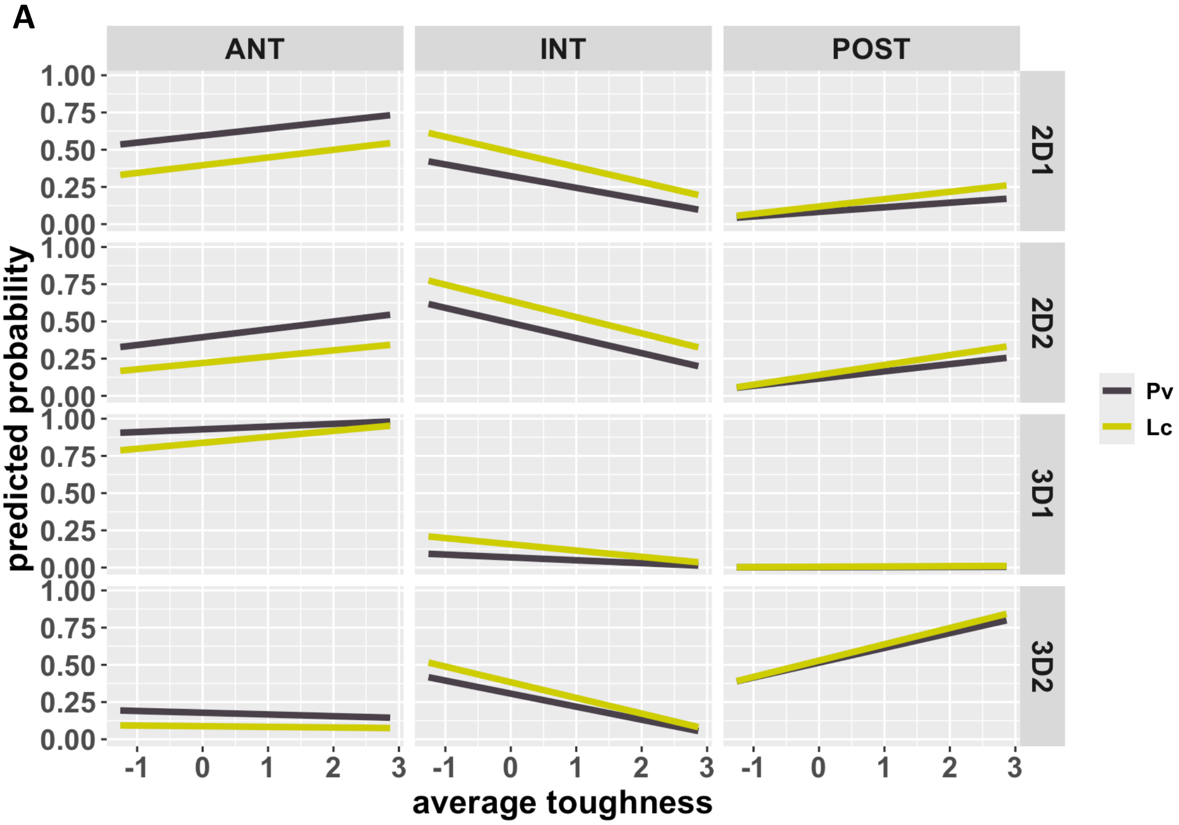
ANT
INT
POST
2D1
2D2
3D1
3D2
predicted probability
1.00
0.75
0.50
0.25
0.00
-1
0
1
2
3
average toughness
Pv
Lc

B

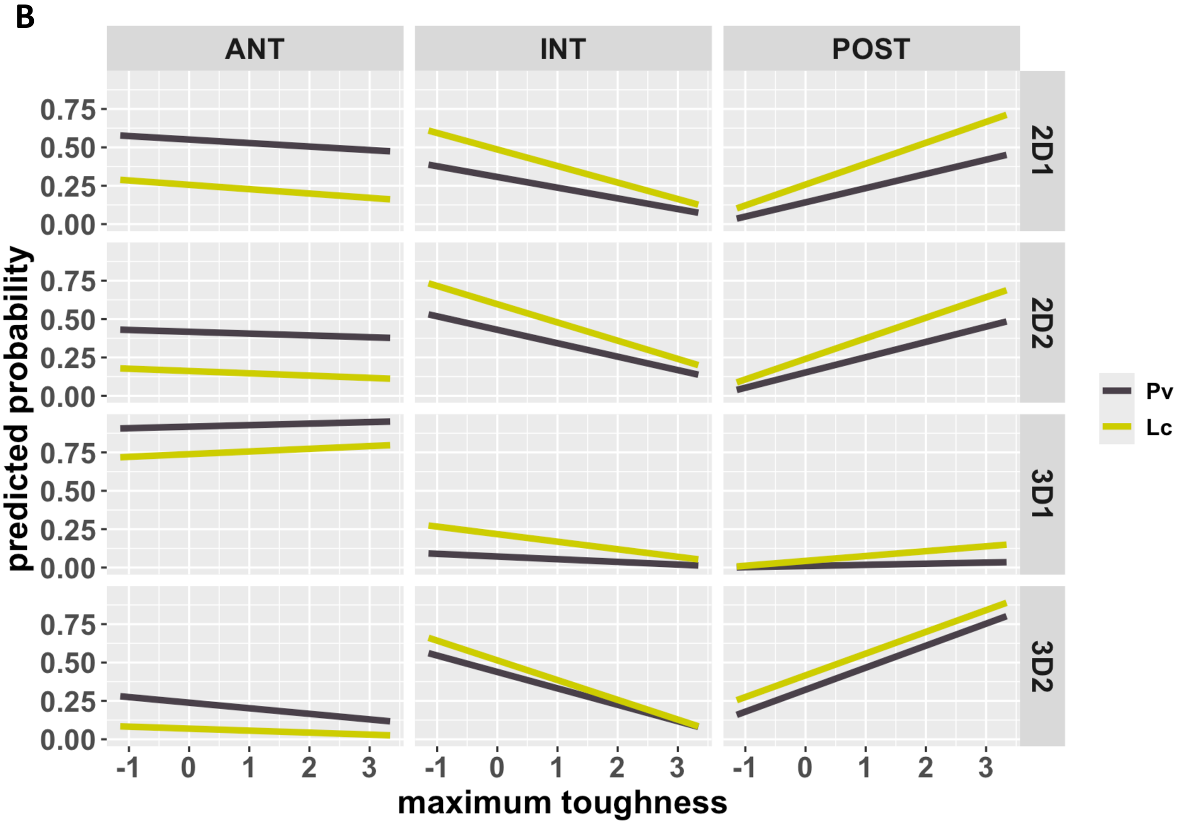
ANT
INT
POST
2D1
2D2
3D1
3D2
predicted probability
0.75
0.50
0.25
0.00
-1
0
1
2
3
maximum toughness
Pv
Lc

FIGURE S5: Contrasts of predicted bite positions by food geometry for models with A) average toughness, R_{av}, and B) maximum toughness, R_{max}, as the covariate. Slopes are estimated for toughness at each bite position with respect to lemur species using the emmeans package. Plots were made with the emmeans function, emmip, then customized in ggplot2. R_{av} and R_{max} have been Z-scaled. ANT, anterior of mouth; INT, intermediate position of postcanine toothrow; POST, posterior-most part of toothrow; 2D1, flat, small food geometry; 2D2, flat, large geometry; 3D1, non-flat, small geometry; 3D2, non-flat, large geometry; Pv, *Propithecus verreauxi*; Lc, *Lemur catta*. See text for further details on toughness values, biting positions, and food geometry.
